# Dissemination interventions to improve healthcare workers’ adherence with infection prevention and control guidelines: a systematic review and meta-analysis

**DOI:** 10.1186/s13012-021-01164-6

**Published:** 2021-10-24

**Authors:** Marcus Tolentino Silva, Tais Freire Galvao, Evelina Chapman, Everton Nunes da Silva, Jorge Otávio Maia Barreto

**Affiliations:** 1 Pharmaceutical Science Graduate Course, University of Sorocaba, São Paulo, Brazil; 2 Faculty of Pharmaceutical Sciences, University of Campinas, Campinas, Brazil; 3 Oswaldo Cruz Foundation, Brasília, Brazil; 4 Faculty of Ceilândia, University of Brasilia, Brasília, Brazil

**Keywords:** Infection prevention and control, Acute respiratory tract infections, Clinical practice guideline, Guideline adherence, Implementation strategies, Implementation outcomes, Health personnel

## Abstract

**Background:**

The COVID-19 pandemic has challenged health systems worldwide since 2020. At the frontline of the pandemic, healthcare workers are at high risk of exposure. Compliance with infection prevention and control (IPC) should be encouraged at the frontline. This systematic review aimed to assess the effects of dissemination interventions to improve healthcare workers’ adherence with IPC guidelines for respiratory infectious diseases in the workplace.

**Methods:**

We searched CENTRAL, MEDLINE, Embase, and the Cochrane COVID-19 Study Register. We included randomized controlled trials (RCTs) and cluster RCTs that assessed the effect of any dissemination strategy in any healthcare settings. Certainty of evidence was assessed using the GRADE approach. We synthesized data using random-effects model meta-analysis in Stata 14.2.

**Results:**

We identified 14 RCTs conducted from 2004 to 2020 with over 65,370 healthcare workers. Adherence to IPC guidelines was assessed by influenza vaccination uptake, hand hygiene compliance, and knowledge on IPC. The most assessed intervention was educational material in combined strategies (plus educational meetings, local opinion leaders, audit and feedback, reminders, tailored interventions, monitoring the performance of the delivery of health care, educational games, and/or patient-mediated interventions). Combined dissemination strategies compared to usual routine improve vaccination uptake (risk ratio [RR] 1.59, 95% confidence interval [CI] 1.54 to 1.81, moderate-certainty evidence), and may improve hand hygiene compliance (RR 1.70; 95% CI 1.03 to 2.83, moderate-certainty). When compared to single strategies, combined dissemination strategies probably had no effect on vaccination uptake (RR 1.01, 95% CI 0.95 to 1.07, low-certainty), and hand hygiene compliance (RR 1.16, 95% CI 0.99 to 1.36, low-certainty). Knowledge of healthcare workers on IPC improved when combined dissemination strategies were compared with usual activities, and the effect was uncertain in comparison to single strategy (very low-certainty evidence).

**Conclusions:**

Combined dissemination strategies increased workers’ vaccination uptake, hand hygiene compliance, and knowledge on IPC in comparison to usual activities. The effect was negligible when compared to single dissemination strategies. The adoption of dissemination strategies in a planned and targeted way for healthcare workers may increase adherence to IPC guidelines and thus prevent dissemination of infectious disease in the workplace.

**Trial registration:**

Protocol available at http://osf.io/aqxnp.

Contributions to the literature

- Research has addressed implementation strategies in healthcare services, but there remains a lack of reliable evidence on specific implementation strategies to support the implementation of IPC guidelines in different contexts.
- These findings contribute to the recognition of the best available evidence and research gaps on the effects of dissemination interventions to improve healthcare professionals' adherence to the IPC guidelines for infectious respiratory diseases in the workplace.
- Interventions to improve adherence to IPC guidelines are relevant to global, national, and local contexts and can help to reduce implementation gaps in the pandemic setting, as well as to prepare for future health emergencies.

## Background

Severe acute respiratory syndrome coronavirus 2 (SARS-CoV-2), which causes coronavirus disease 2019 (COVID-19), continues to spread globally since the declaration of the COVID-19 pandemic in 2020 [1–6]. Knowledge about transmission, signs and symptoms, and prognostic factors has evolved rapidly and improved decision-making for this global threat [6]. Governments have implemented different non-pharmacological strategies to control person-to-person transmission, such as use of masks, quarantine, and social distancing, which has led to control of the spread [3, 7]. A combination of these strategies seems to be key for their success, which continues to be dynamic with emerging variants, changes in policies, and disease waves—within and across countries—, which increases the disease burden [8]. At the same time, an unprecedented global effort has also enabled the development of high-efficacy vaccines [9].

At the frontline of the pandemic, healthcare workers are considered at high risk of exposure [10]. Several factors increase this risk, such as prolonged exposure to large numbers of infected and asymptomatic people, inadequate personal protection due to shortage of personal protective equipment or respirator reuse and extended use policies, and insufficient training for infection prevention and control (IPC) [11]. In China, 4% of COVID-19 cases were in healthcare workers [12], accounting for 30% of total hospitalizations related to COVID-19 in Wuhan during January 2020 [13]. By the end of the first quarter of 2020, COVID-19 infections were estimated to be between 10 and 20% among healthcare workers in Italy [12].

Since the healthcare setting seems to play an important role in the spread of the disease [14], achieving high compliance with IPC measures requires changes in behavior and changes in the workplace. There are still gaps in the processes of translating the best evidence into practice. In this context, it is important to know which implementation strategies based on dissemination interventions are the most effective to improve healthcare workers’ adherence to IPC recommendations [15–17].

Health-related information dissemination is primarily focused on communicating research results, targeting and tailoring the findings and messages to an appropriate audience (‘help to make it happen’) [18, 19]. Dissemination also involves an active and personalized process, a necessary step for knowledge adoption and implementation in the field of public health or clinical practice [20].

Implementation strategies designed for healthcare workers include a number of different interventions. Such interventions involve various components to be delivered through a variety of modalities and in different contexts. Due to the vast set of interventions aiming to disseminate guidelines or recommendations in health services, the Cochrane Effective Practice and Organization of Care (EPOC) taxonomy [21] is a practical way to identify implementation strategies targeted at workers and designed to improve adherence to IPC guidelines. Implementation strategies are targeted at healthcare organizations and mainly include audit and feedback, patient or provider education, reminders, mentoring, etc. [21].

Implementation strategies related to dissemination must be fostered in health services to support behavior changes of healthcare professionals in the workplace aiming at increasing adherence to guidelines for IPC [17]. These strategies can improve the delivery, practice, and organization of healthcare services in different scenarios [22, 23].

Behavior change of healthcare providers may require complex approaches and several factors could influence adherence to IPC guidelines when managing respiratory diseases, for instance, factors related to the message itself and the way of disseminating it, factors related to organizational culture, and other contextual factors [17, 23, 24]. These and other factors should be considered when deciding to implement different dissemination strategies in healthcare settings [25, 26].

In this scenario, we reviewed the current literature to assess the effects of dissemination interventions to improve healthcare workers’ adherence to IPC guidelines for respiratory infectious diseases in the workplace.

## Methods

This systematic review was conducted following the Cochrane handbook for methods [27] and the reporting adhered to the Preferred Reporting Items for Systematic Reviews and Meta-Analysis (PRISMA) 2020 statement [28]. A previous protocol was developed and published in the Open Science Framework repository (http://osf.io/aqxnp).

### Searches

We searched Cochrane Central Register of Controlled Trials (CENTRAL; 2020, Issue 9) in the Cochrane Library (searched on 23 September 2020); MEDLINE (via Ovid; 1946 to 23 September 2020); Embase (via Ovid; 1974 to 23 September 2020); and Cochrane COVID-19 Study Register (February 2020 to 23 September 2020; http://covid-19.cochrane.org). We screened the references of related Cochrane systematic reviews and the list of references of the included studies.

An information specialist conducted our search of the literature, which was revised by a content expert. Complete information on the search strategies is available in the protocol. We limited the searches to randomized controlled trials (RCTs) and no other limits were applied. Search outputs were imported into Covidence platform (www.covidence.org) to remove duplicates and perform further review steps.

### Selection process

The team of review authors (MTS, TFG, EC, ENS, JOMB) in pairs and independently screened titles and abstracts at Covidence platform. After screening the first 100 studies, the team met to assess disagreements and adjust the selection process. We resolved disagreements by consensus. The same process was applied to select studies in full text that were considered eligible based on title and abstract screening.

### Study quality assessment

We used the Cochrane risk-of-bias tool for RCT version 1 [29], integrated with Covidence [30], to assess the included studies (dual; second reviewer checks all judgements). We judged the risk of bias as “low,” “high,” or “unclear” and provided support for judgement of the following items: sequence generation, allocation concealment, blinding of participants and personnel, blinding of outcome assessors, incomplete outcome data, selective outcome reporting, and other sources of bias. We adopted “unclear risk” only in cases of lack of information about the methods.

### Data extraction strategy

All authors extracted data from the studies (MTS, TFG, EC, ENS, JOMB) using a customized form in Covidence, which were cross-checked by a second author (MTS, TFG).

We collected characteristics of the studies (author, year of research, country, setting, study design, inclusion and exclusion criteria, sponsorship source, conflicts of interest), characteristics of the study participants, description of the interventions, and results.

### Data synthesis and presentation

We sought data for adherence to IPC guidelines in each intervention group assessed in the studies according to the nature of the data. We grouped the outcomes of similar enough studies according to the intervention and longest available follow-up. For vaccine uptake, we collected the number of healthcare workers vaccinated and the total number of personnel assessed in each group. Hand hygiene compliance data relied on the number of hand hygiene actions by all hand hygiene opportunities (before patient contact, before aseptic task, after body fluid exposure, after patient contact, after contact with patient surroundings). Knowledge about IPC data was based on the number of individuals assessed and measured for knowledge in each group (mean and standard deviation of the test score or score improvement and interquartile range).

We calculated the mean differences (MD) for knowledge on IPC and risk ratios (RR) of vaccination uptake and hand hygiene compliance outcomes along with 95% confidence intervals (CI). Outcome effect of each intervention was assessed in comparison to usual activities or other strategies. As studies’ interventions relied on multiple dissemination interventions, effects were presented separately into “combined strategies vs. usual activities” and “combined strategies vs. single strategies.” We adopted random-effects meta-analysis for all outcomes [27], considering the outcomes as related but slightly divergent intervention effects. For the cluster RCTs included, we calculated the design effect using the intracluster correlation coefficient, the number of clusters and the average sample size of each cluster. We calculated the RR by entering the sample size and the number of results adjusted by the design effect [29]. We used Stata (version 14.2) to calculate all meta-analyses. When meta-analysis was not feasible, we synthesized the results narratively. We assessed the presence of heterogeneity by inspecting forest plots and calculated the *I*^2^ statistic and Chi^2^ test. In visually discrepant results in the forest plots distribution, we considered as substantial heterogeneity results with significant Chi^2^ test (*p* < 010) and *I*^2^ statistic > 50% [27].

### Evidence of effectiveness

We judged available outcomes (vaccination uptake, hand hygiene compliance, and knowledge) using the Grading of Recommendations Assessment, Development and Evaluation (GRADE) approach to assess the certainty of the evidence in its five domains: limitations, indirectness, imprecision, inconsistency, and other factors [31]). We rated the certainty of the evidence of each outcome as “very low,” “low,” “moderate,” or “high” and prepared evidence profiles and summary of findings tables of the effects of combined strategies in comparison to the controls (usual activities or single strategies).

## Results

### Review statistics

**Fig. 1:**
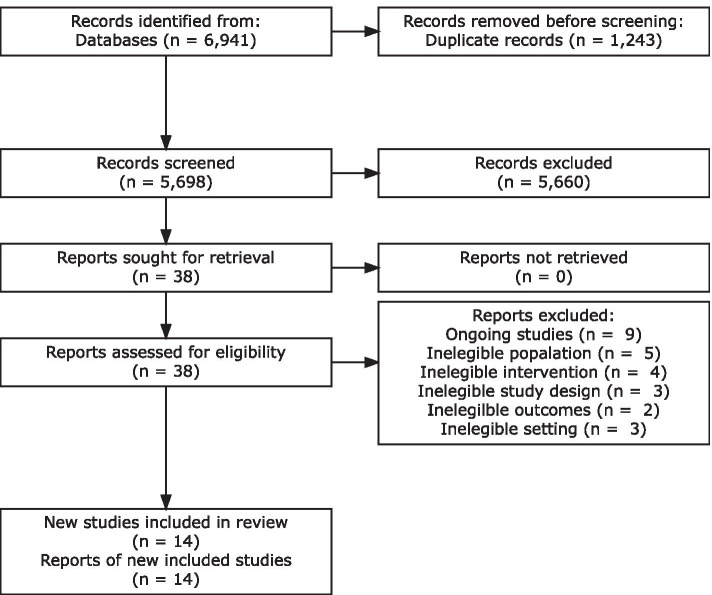
Process of selection and inclusion of studies

Out of 6941 retrieved records and 2 additional records identified through other sources, we screened 5698 unique records after duplicate removal based on title and abstracts. We assessed full text of 38 studies and included 14 studies [32–45] in this systematic review and meta-analysis (Fig. 1).

We excluded 15 full-text studies. Five had an ineligible population [46–50], four had an ineligible intervention [51–54], three had an ineligible study design [55–57], two had ineligible outcomes [58, 59], one occurred in offices and thus had an ineligible setting [60]. Nine studies were ongoing up to the conclusion of this review [61–69], of which four started in 2020 [64–67]. Two trials were registered in 2007 and 2009 and remained “ongoing” in their protocols [68, 69]. All of them assessed combined dissemination strategies to improve IPC for healthcare workers, including education, training, audit and feedback, positive deviance, a voice enabled virtual assistant (Amazon Alexa device), gamification, and evidence-based telehealth [61–67].

### Study characteristics

We included seven parallel RCTs and seven cluster-RCTs, conducted from 2004 to 2020 and funded mainly with research sponsorship (Table 1). Over 65,370 healthcare workers of all categories were assessed for infection prevention and control adherence outcomes that included influenza vaccination uptake, hand hygiene compliance, and knowledge on infection prevention and control. Two studies did not state the number of healthcare workers assessed, just the number of opportunities for hand hygiene [37, 44].

**Table 1:** Characteristics of included studies

Study	Year	Design	Participants	Sample (*n*)	Interventions	Comparator	Outcomes	Funding	Declaration of interest
Combined strategies versus usual activities									
Borgey 2019 [32]	2014–2015	Cluster RCT	Healthcare workers in contact with patients	1336	Educational materials, opinion leaders, vaccine	Usual activities	Vaccination uptake	None	None
Ho 2012 [33]	2009–2010	Cluster RCT	Healthcare workers in contact with patients	810	WHO multimodal hand hygiene improvement strategy	Usual activities	Hand hygiene compliance	Centre for Health Protection	None
Jeihooni 2018 [34]	2016	RCT	Nurses in contact with patients	120	Educational materials and meetings	Usual activities	Knowledge on hand hygiene	None	None
Lehmann 2016 [35]	2014	RCT	Healthcare workers	122	Educational materials and meetings, vaccination appointment and reminder	Usual activities	Vaccination uptake	Abbot Health Care Products B.V.	None
Martin-Madrazo 2012 [36]	2009	Cluster RCT	Healthcare workers	170	WHO multimodal hand hygiene improvement strategy	Usual activities	Hand hygiene compliance	Ministry of Health of Spain	None
Mertz 2010 [37]	2007–2008	Cluster RCT	Healthcare workers in contact with patients	a	WHO multimodal hand hygiene improvement strategy, opinion leaders	Usual activities	Hand hygiene compliance	Physicians’ Services Incorporated Foundation of Ontario, Swiss National Service Foundation	None
Riphagen-Dalhuisen 2013 [38]	2008–2010	RCT	Healthcare workers in contact with patients	50,351	Educational materials and meetings, vaccine	Usual activities	Vaccination uptake	The Netherlands Organization for Health Research and Development	None
Rothan-Tondeur 2011 [39]	2005–2006	RCT	Healthcare workers in contact with patients	2874	Educational materials and meetings	Usual activities	Vaccination uptake	None	Five out of 8 authors are consultants or received grants from pharmaceutical industry
Yeung 2011 [40]	2007	Cluster RCT	Healthcare workers in contact with patients	188	WHO multimodal hand hygiene improvement strategy	Usual activities	Hand hygiene compliance	Chinese University of Hong Kong and Vickmans Laboratories	None
Combined strategies versus usual single strategies									
Doratotaj 2008 [41]	2004–2005	RCT	Healthcare workers in contact with patients	800	Educational materials and meetings, vaccine	Educational materials	Vaccination uptake	The Cleveland Clinic Foundation	None
Huis 2013 [42]	2008–2009	Cluster RCT	Nurses in contact with patients	886	WHO multimodal hand hygiene improvement strategy, local opinion leaders	WHO multimodal hand hygiene improvement strategy	Hand hygiene compliance	The Netherlands Organization for Health Research and Development	None
Schmidtke 2020 [43]	2018–2019	RCT	Healthcare workers in contact with patients	7540	Educational materials, reminders, performance monitoring	Reminders	Vaccination uptake	National Institute for Health Research	None
Stewardson 2016 [44]	2009–2014	Cluster RCT	Healthcare workers in contact with patients	a	WHO multimodal hand hygiene improvement strategy, enhanced feedback, performance monitoring	WHO multimodal hand hygiene improvement strategy	Hand hygiene compliance	Swiss National Science Foundation	None
Suppan 2020 [45]	2020	RCT	Healthcare workers in contact with patients	173	Educational material and game	Educational material	Knowledge on IPC	None	None

*Note*: *IPC* infection prevention and control, *RCT* randomized controlled trial, *WHO* World Health Organization

^a^Number of healthcare workers not reported (opportunities of hand hygiene were measured)

**Fig. 2:**
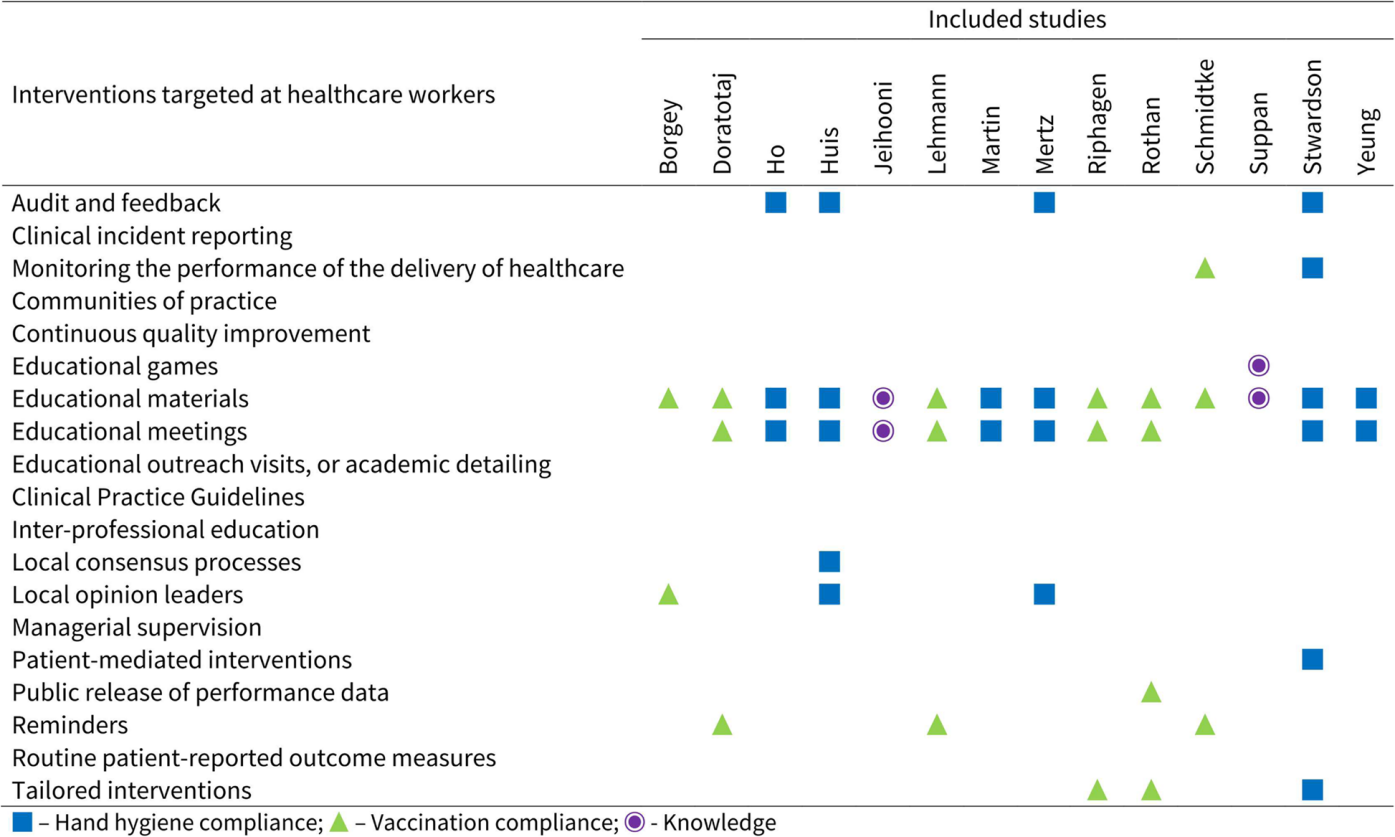
Interventions assessed by included studies

Figure 2 displays the interventions assessed by included studies. All studies based their dissemination of implementation strategies on educational interventions, including materials, meetings, and games [32–35, 37–45, 70]. Six studies that assessed hand hygiene compliance [33, 37, 40, 42, 44, 70] used adapted versions of the WHO multimodal hand hygiene improvement strategy, which includes provision of products and infrastructure for hand hygiene, education, observation and feedback, reminders in the workplace, and creation of a safety culture. Three studies [38, 39, 44] conducted surveys and focus group sessions to tailor their dissemination interventions. Monitoring the performance of the delivery of healthcare was employed in two [33, 44], and audit and feedback in four studies [33, 37, 42, 44]. Patient-mediated interventions were used in one of the experimental groups in one study [44], and public release of performance data was part of the intervention in another [39].

Most studies were held in hospitals [34, 37–39, 41–45], three in nursing homes [32, 33, 40], one in primary healthcare center [70], and one in a reference clinic for chronic diseases [35]. Nine studies took place in Europe, three in Asia, and two in America (Table 1).

### Study quality assessment

**Fig. 3:**
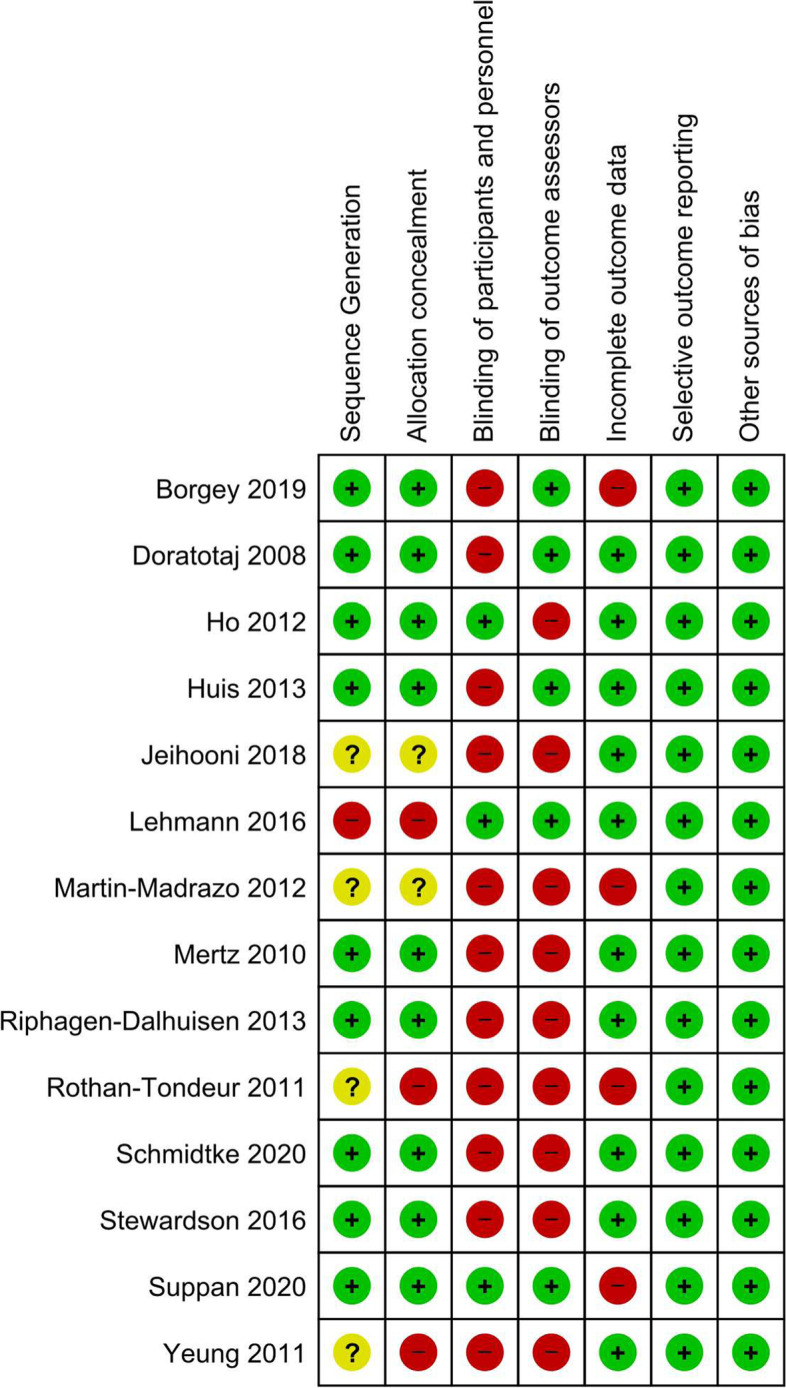
Risk of bias of included studies

The main biases of the studies were lack of blinding of participants, personnel, and outcomes assessors (Fig. 3). Sequence generation, allocation concealment, and incomplete outcome data affected over one quarter of studies. No study was free from risk of bias (Fig. 3).

Nine studies used adequate methods for random sequence generation [32, 33, 37, 38, 41–45] and were at low risk of selection bias. Four did not describe the randomization method and were classified as unclear [34, 39, 40, 70]. One study [35] relied on an alphabetical list of the workers, leading to high risk of bias in randomization.

Nine included studies adequately concealed the allocation [32, 33, 37, 38, 41–45], two were not clear about this process [34, 70], and three did not conceal the allocation [35, 39, 40], regarded as high risk of selection bias.

Three studies adequately blinded participants and personnel and were at low risk of bias [33, 35, 45]. It was not possible to blind participants and the personnel to their group due to the nature of interventions in 11 studies classified as high risk of performance bias [32, 34, 37–44, 70]. Nine studies did not blind their outcomes assessors to the intervention and we rated as high risk of detection bias [33, 34, 37–40, 43, 44, 70]. Five RCTs blinded the outcomes assessors, considered as low risk of detection bias [32, 35, 41, 42, 45].

Four studies were at high risk of attrition bias [32, 39, 45, 70] due to losses of facilities or participants during follow up. The other 10 studies had no problem regarding incomplete data; thus, we considered them to be at low risk of attrition bias [33–35, 37, 38, 40, 41, 43, 44]. We assessed all studies as having a low risk of reporting bias, since they reported the outcomes as described in their protocol or methods.

### Vaccination uptake

**Fig. 4:**
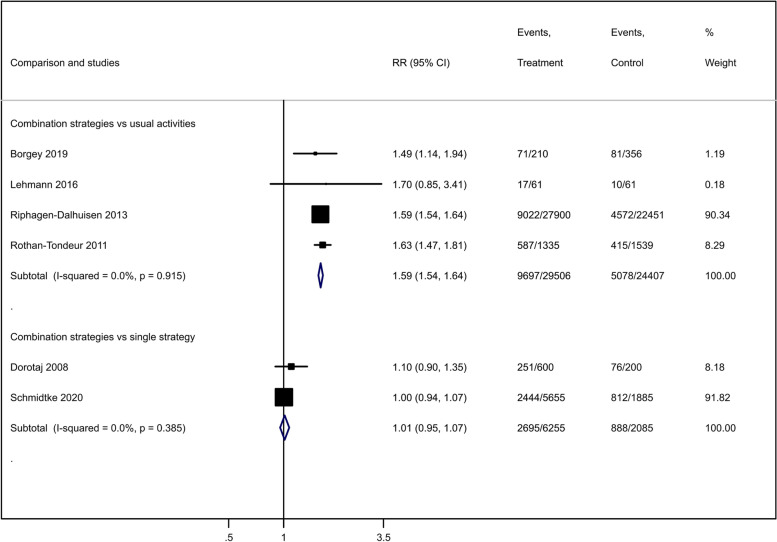
Effect of combined dissemination strategies compared to usual activities on healthcare workers' influenza vaccination uptake

Combined dissemination strategies improved the influenza vaccination uptake compared to usual activities (RR 1.59, 95% CI 1.54 to 1.81; 4 studies [32, 35, 38, 39], 53,913 participants; *I*^2^ = 0%; moderate-certainty evidence; Fig. 4). We downgraded the certainty of evidence by one level for study limitations (Table 2).

**Table 2:** GRADE evidence profile for vaccination uptake, hand hygiene compliance and knowledge

Certainty assessment							No. of patients		Effect		Certainty
No. of studies	Study design	Risk of bias	Inconsistency	Indirectness	Imprecision	Other considerations	Combined strategies	Comparison	Relative (95% CI)	Absolute (95% CI)	
Vaccination uptake (combined strategies vs usual activities)											
4	RCT	Serious ^a^	Not serious	Not serious	Not serious	None	9697/29,506 (32.9%)	5078/24,407 (20.8%)	RR 1.59 (1.54 to 1.64)	123 more per 1000 (from 112 more to 133 more)	⨁⨁⨁◯ Moderate
Vaccination uptake (combined strategies vs single strategies)											
2	RCT	Serious ^a^	Not serious	Not serious	Serious ^b^	None	2695/6255 (43.1%)	888/2085 (42.6%)	RR 1.01 (0.95 to 1.07)	4 more per 1000 (from 21 fewer to 30 more)	⨁⨁◯◯ Low
Hand hygiene compliance (combined strategies vs usual activities) (assessed with: opportunities for hand hygiene)											
4	RCT	Serious ^a^	Not serious	Not serious	Not serious	None	529/1137 (46.5%)	310/997 (31.1%)	RR 1.70 (1.03 to 2.83)	218 more per 1000 (from 9 more to 569 more)	⨁⨁⨁◯ Moderate
Hand hygiene compliance (combined strategies vs single strategies) (assessed with: opportunities for hand hygiene)											
2	RCT	Serious ^a^	Not serious	Not serious	Serious ^b^	None	1350/1992 (67.8%)	726/1366 (53.1%)	RR 1.16 (0.99 to 1.36)	85 more per 1000 (from 5 fewer to 191 more)	⨁⨁◯◯ Low
Knowledge (combined strategies vs usual activities)											
1	RCT	Very serious ^a,c^	Not serious	Serious ^d^	Not serious	None	60	60	-	MD 4.1 higher (3.39 higher to 4.81 higher)	⨁⨁◯◯ Low
Knowledge (combined strategies vs single strategies)											
1	RCT	Serious ^e^	Not serious	Serious ^d^	Serious ^b^	None	Score improvement 17% (IQR 8 to 33%) versus 8% (IQR 8 to 33%); *p* = 0.27				⨁◯◯◯ Very low

Note: *CI* confidence interval, *RCT* randomized controlled trial, *RR* risk ratio, *MD* mean difference

^a^Problems in blinding and allocation unconcealed

^b^Non-significant confidence interval

^c^Sequence generation not adequate

^d^Indirect outcome for adherence to infection, prevention, and control measures

^e^Incomplete outcome data

Combined dissemination strategies may have little effect or no effect on influenza vaccination uptake, compared to a single dissemination strategy (RR 1.01; 95% CI 0.95 to 1.07; 2 studies [41, 43]; 8340 participants; *I*^2^ = 0%; low-certainty evidence; Fig. 4). We downgraded the certainty of evidence by two levels for limitations and imprecision (Table 2).

### Hand hygiene compliance

**Fig. 5:**
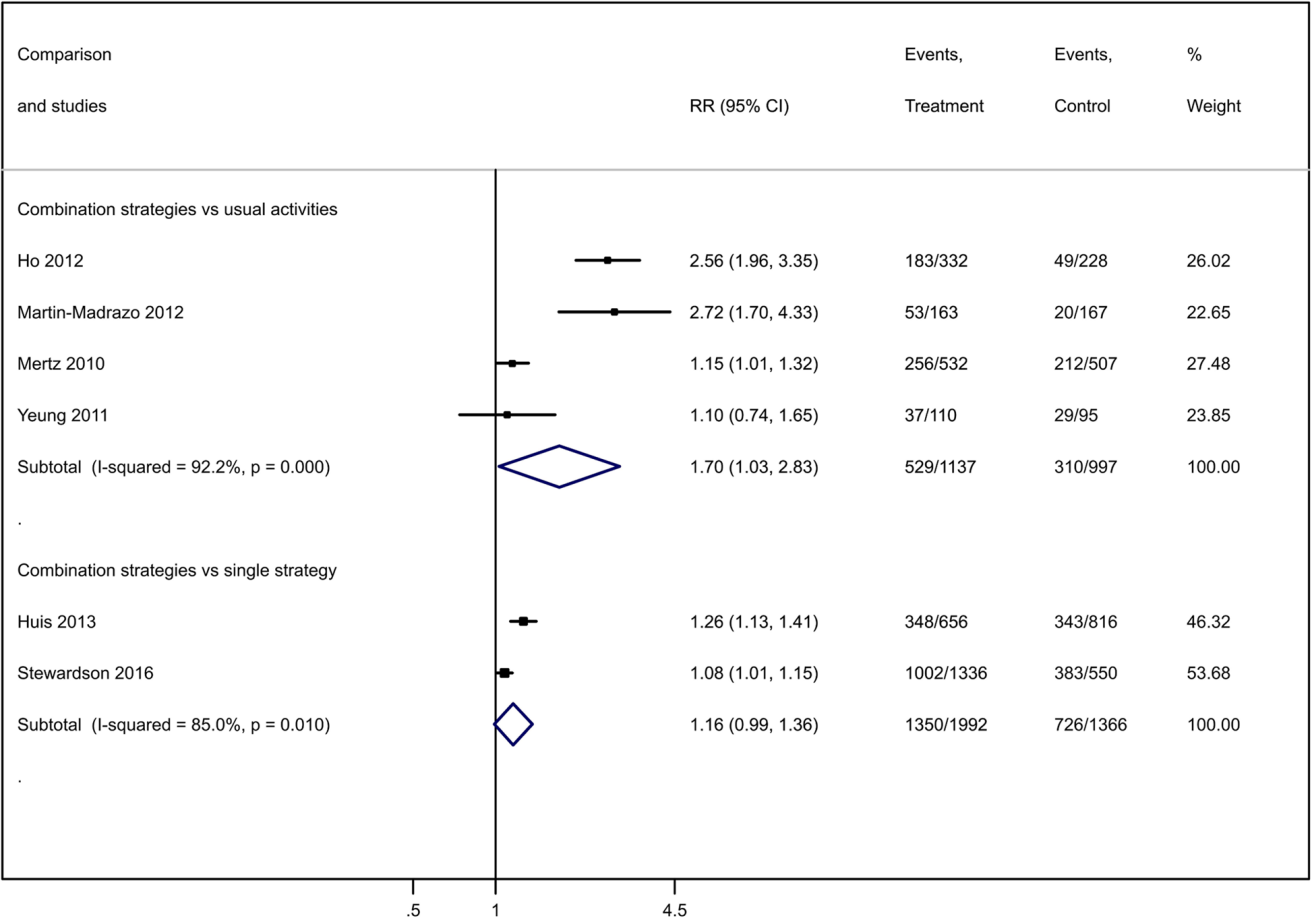
Effect of combined dissemination strategies compared to usual activities on healthcare workers' hand hygiene compliance

Combined dissemination strategies compared to usual activities improved healthcare workers’ hand hygiene compliance (RR 1.70; 95% CI 1.03 to 2.83; 4 studies [33, 37, 40, 70]; 2134 hand hygiene opportunities; *I*^2^ = 92.2%; moderate-certainty evidence; Fig. 5). We downgraded the certainty of evidence by one level for study limitations (Table 2). As directions of studies’ effects were similar, serious heterogeneity was disregarded. We did not find any factor (year, design, settings, participants, sample size, intervention, or funding) among these studies that explained the statistical heterogeneity.

Combined dissemination strategies may have little effect or no effect on hand hygiene compliance, compared to a single dissemination strategy (RR 1.16; 95% CI 0.99 to 1.36; 2 studies [42, 71]; 3358 hand hygiene opportunities; *I*^2^ = 85%; low-certainty evidence; Fig. 5). Homogeneity in the directions of studies’ effects led us to disregard serious inconsistency. We downgraded the certainty of evidence by two levels due to study limitations and imprecision (Table 2).

### Knowledge

One study assessed whether educational materials and meetings compared to usual activities would improve knowledge about preventive behaviors against healthcare-associated infections in hospital nurses [34]. The researchers assessed knowledge in the pre-intervention, post-intervention and 4 months later, using a questionnaire with a scale ranging from 0 (insufficient knowledge) to 10 (adequate knowledge). These combined dissemination strategies improved healthcare workers’ knowledge of preventive behaviors on IPC, compared with usual activities (MD 4.10; 95% CI 3.39 to 4.81 in post-intervention; MD 4.1; 95% CI 3.36 to 4.84 in 4 months later; 120 participants; very low-certainty evidence). Due to very low certainty evidence—downgraded by three levels for study limitations, indirectness, and imprecision—, we are uncertain of this effect (Table 2).

One study assessed whether the educational game plus the pre-hospital COVID-19 guidelines compared to the guideline alone would improve knowledge about the use of protective equipment [45]. The researchers measured knowledge using an online survey about the choice of personal protective equipment in short clinical scenarios on a scale of percentage of correct answers (0 to 100%). We are uncertain if combined dissemination strategies impact on healthcare workers’ knowledge of use of protective equipment, compared to a single strategy (17% IQR 8, 33% versus 8% IQR 8, 33%; *p* = 0.27; 173 participants; very low-certainty evidence). We downgraded the certainty of evidence by three levels for study limitations, indirectness, and imprecision (Table 2).

## Discussion

Combined strategies compared to usual activities improved the influenza vaccination uptake (moderate-certainty evidence), hand hygiene compliance (low-certainty evidence), and knowledge (very low-certainty evidence). When compared to single strategies, combined interventions did not improve vaccination uptake (low-certainty evidence), hand hygiene compliance (low-certainty evidence), and knowledge (very low-certainty evidence).

This systematic review covered a diverse set of drivers that could improve the IPC practices for respiratory infectious diseases in healthcare workers, such as vaccination, hand hygiene, and knowledge about infection prevention, but we did not find any RCT that focused especially on the implementation of IPC guidelines. In addition, we have not provided subgroup analyses and equity considerations of the assessed dissemination interventions because the studies have not stratified their results by gender, age groups, or healthcare workers’ categories.

Despite digital media have wide availability, few studies employed strategies for dissemination using electronic means. Healthcare workers, including those who have worked in the pandemic, are familiar with electronic tools [72]. Strategies that use this type of dissemination could be leveraged to improve the compliance with protocols and guidelines for IPC among healthcare workers, and many challenges have already been recognized [73]. Digital competence may vary depending on the setting and low and middle-income countries' contexts, which may require specific approaches to address gaps to apply these strategies [74].

Analyses by professional category were not feasible also considering that the included studies covered a wide range of healthcare workers, such as doctors, nurses, therapists, assistants, among others, assessed in settings from primary to tertiary care. The included studies assessed dissemination strategies in settings with hospitalizations and long-term care units, with intense contact with patients that raises the risk of spread of infection.

Compared to no intervention, combined dissemination strategies increased the uptake of vaccination, hand hygiene compliance, and knowledge about infection prevention. While combined strategies showed to be effective, it is unclear whether they would be superior to single intervention strategies. To maintain the best balance in the dissemination strategy, decision-makers should monitor the impact along with the implementation and consider equity issues, in order to include considerations about, for example, the different pre-existing socioeconomic and cultural conditions that influence disparities related to risks and health outcomes in the pandemic. The improvement of combined intervention when compared to no intervention and its low effect when compared to a single intervention were also observed by studies that focused on strategies to support the dissemination of guidelines [75–77].

We hypothesize that a single dissemination strategy can potentially improve healthcare workers’ adherence to good practices to prevent infections and may be a good starting point to change behavior. Despite superior results of combined strategies in comparison to single ones in present review, advantages of single interventions, when compared to multifaceted interventions, have been previously observed [24]. In a pandemic, rapid and specific changes would potentially bring positive results with less use of resources and stressful workload. Future research should evaluate these single interventions compared to usual care in order to confirm the effectiveness of these interventions, which would have lower cost and better viability.

Workers may feel insecure when local guidelines are long, unclear, or do not correspond to national or international guidelines [17]. The level of support received interferes with healthcare workers’ responses to follow IPC guidelines, as some strategies can lead to a greater workload. Clear communication about the guidelines and proper training are also essential for improvement. Altogether, these factors can influence whether healthcare workers follow the guidelines or not [17]. Effective dissemination strategies are thus central to strengthening the process of implementing IPC guidelines, and should be prioritized by decision-makers, especially in low-resource settings [78].

## Conclusions

Compared to no intervention, combined dissemination strategies increased healthcare workers’ vaccination uptake, hand hygiene compliance, and knowledge about infection prevention. When compared to single dissemination strategies, the effect was modest or null. Further research should focus on assessing the effectiveness of single interventions compared to usual practices. The results seem to be favorable to the use of educational strategies combined with other non-educational dissemination strategies, such as audit and feedback. Dissemination strategies may increase adherence to IPC guidelines for healthcare workers management of respiratory diseases and thus prevent their dissemination in the workplace.

## Data Availability

The data and materials supporting the conclusions of this article are available at http://osf.io/aqxnp.

## Abbreviations

CI: Confidence interval
COVID-19: Coronavirus disease 2019
EPOC: Cochrane Effective Practice and Organization of Care
GRADE: Grading of Recommendations Assessment, Development and Evaluation
IPC: Infection prevention and control
IQR: Interquartile range
MD: Mean differences
PRISMA: Preferred Reporting Items for Systematic Reviews and Meta-Analysis
RCT: Randomized controlled trial
RR: Risk ratio
SARS-CoV-2: Severe acute respiratory syndrome coronavirus 2
